# Supplementary material for: Bacteria face trade-offs in the decomposition of complex biopolymers
Source: PLoS Comput Biol. 2024 Aug 8;20(8):e1012320. doi: 10.1371/journal.pcbi.1012320 (PMC11364420; doi:10.1371/journal.pcbi.1012320)
Supplement: Attachment — Submitted filename: response_plos_final.pdf [file pcbi.1012320.s003.pdf]

Dear Tobias Bollenbach,

Thank you for the referee reports, which we have carefully taken into account.
We thank both referees for their thoughtful and constructive comments, and appreciate their general positive response. Our detailed reply to each reviewer's comments (quoted from the report and stated in bold) including a description of which changes we made to the manuscript are given below.
On behalf of the authors,

Ksenia Guseva

Reviewer #1: Review by Stefano Manzoni, Stockholm University

The manuscript by Guseva and co-authors investigates advantages and disadvantages of producing different types of enzymes in microbial populations and communities. This topic is timely as we strive to capture the decomposition process in more mechanistic ways in mathematical models. The proposed model setup is sound and the results are interesting. Despite the complexity of the model and interpretation of some of the results, the model and methods in general are explained in a very clear way. Figures provide an information-rich—but still accessible—overview of the findings. I also liked the mix of literature review to set the stage and context, and theoretical developments. It is an unusual combination, but I found it effective. I have some comments that might help linking the current study with other recent ones, and some suggestions on the model description. Detailed suggestions are listed at the end.

Main comments

1) Novelty and link to recent literature. The mathematical model proposed here is quite novel, but recent work has tackled similar questions and should be considered to place the advances and results into context. Weverka et al. (2023, https://doi.org/10.1016/j.soilbio.2023.109161) investigated trade-offs in enzyme production from the point of view of enzyme allocation and costs. Sainte-Marie et al. (2021, https://doi.org/10.1038/s41467-021-21079-6) have already integrated endo- and exo-enzymes in their decomposition model. They also consider a range of substrates, while you focus on more or less complex carbohydrates. What are the differences between your approach and theirs? What are the methodological novelties of this work?

We thank the referee for pointing out the two additional references we were not aware of. We have added citations of both papers to the introduction. Weverka et al. (2023) simulated the negative impact of chemical diversity on microbial growth using a theoretical model. However, contrary to our model, theirs does not incorporate depolymerization. Instead, the authors consider the assimilation and internal processing of multiple substrates by their corresponding enzymes (one enzyme per substrate), assuming that microorganisms would only invest in substrate assimilation if they receive a return on this investment. The work compares three microbial strategies: sequential breakdown of substrates one after the next, simultaneous assimilation of all substrates at the same time, and repartition of substrates among members of a community.

We incorporate a citation to this work into our introduction, the line 13 now reads:

“Recently, using a theoretical model Weverka et al (2023) showed that chemodiversity of the organic matter pool can hinder its assimilation by microbes.”

The paper by Sainte-Marie et al. (2021) indeed provides an alternative framework to include depolymerization of a complex substrate in the context of a large-scale model of soil organic matter turnover. However, our approach differs from theirs in two fundamental aspects: we explicitly incorporate enzyme kinetics into our model, and we propose an exact framework to model depolymerization considering discrete polymer size classes. We provide additional details of main differences below.

Firstly, the approach from Sainte-Marie et al. (2021) contrary to ours does not include enzymes explicitly, and microbial biomass is used as proxy for enzyme concentrations. As a consequence they also do not consider Michaelis Menten kinetics. Although at large scales such approximation could be valid, on small scales of microbial consortia and for our research question such approach wouldn’t be adequate. Secondly, the approach to model fluxes between different sizes of polymers (fragmentation dynamics) of Sainte-Marie et al. (2021) also differs from ours. They use a well known approximation where the discrete polymer size distribution is replaced by a continuous one. While such approximation is numerically advantageous, it is only well defined for the case of very long chains. More subtle differences can be also noticed in the choice of fragmentation distribution function (kernel in Eq. (6) in Sainte-Marie et al. (2021)) and Eq.(6) in our SI.

We reference the work of Sainte-Marie et al. (2021) in the introduction, line 78 now reads:

“ Despite its importance, a detailed inclusion of multiple steps of depolymerization process into current microbial models [17, 18] or into the models of organic matter decomposition in general [19–22] remains lacking. A notable effort in this direction was made by Sainte-Marie et al. (2021) [23], who included a continuous framework (which approximates the discrete polymer size distribution by a continuous one) to incorporate mass flow between different polymer size classes in a model of soil organic matter turnover. However, their approach still lacks an explicitly inclusion of enzyme (Michaelis-Menten) kinetics and is well-defined only in the presence of very long chain molecules [24].”

In summary, our model is the first model which incorporates all steps of depolymerization process, modeling the mass flux between pools of different polymer size classes (from the large chains towards monomers) in an exact form, and which explicitly models the extracellular enzymes and their reaction with the substrate by Michaelis Menten kinetics adapted to this depolymerization framework. Our approach allows to model the exchange of nutrients and public goods within microbial communities, and model cooperation and competition among microbes that arise during degradation of complex substrates.

2) Fate of depolymerization products. L267-268: diffusion-driven losses are actual losses only with respect to a microbe/microbial colony, but they might be gains for microbes in other grid cells (as noted in L330). Advection is a much more powerful mechanism for removal of dissolved

organic matter. But there are other mechanisms that would act a bit like diffusion—removing depolymerization products from the vicinity of microbes. For example, depolymerization products can be stabilized on soil minerals or trapped into stable aggregates. While the kinetics of these processes are not dependent only on solute concentrations, but also on availability of mineral surfaces/dynamics of aggregates, I would still discuss their potential role in the context of your findings.

We thank the referee for the observation, indeed the sequestration of organic compounds by mineral surfaces could be an additional factor of loss experienced by microorganisms or their colony.

We added a short discussion about mineral-organic matter interactions to the discussion, the line 364 now reads:

"On the other hand all these members of the colony have to compete with additional processes for the uptake of small organic molecules. One of such processes in soils is the adsorption of diffusing molecules on mineral surfaces or other forms of immobilization of organic material, for example by aggregate formation (Kleber et al 2021). "

3) Model description. Please see below in the detailed comments some suggestions. In particular, I would suggest double-checking units and reporting units (and definitions) for all symbols in a separate table.

We thank the referee for carefully reviewing the model description, including the SI. As suggested, we double-checked the units (please see our responses below) and incorporated a new table into the SI containing definitions and units for all symbols.

Detailed comments

We thank the referee for the corrections and suggestions, all were incorporated into the manuscript. We also updated Fig. 5 to allow for larger fonts of the labels.

L21: what are the time scales involved in this example from the literature? are they comparable to time scales relevant in this work?

The work of Hehemann et al. does not present these timescales. Their study is grounded in phylogenetic analyses, indicating that each specific set of degradation-related genes characterizing a taxon results from rapid horizontal gene transfer. Additionally, the study links the presence/absence of these genes with particular environments where the populations were discovered. To prevent misunderstanding, the new line 22 now reads:

"*Hehemann et al showed evidence that through adaptive radiation a population of closely related bacteria has subdivided into specialized ecophysiological types adapted to the given environmental opportunities…*"

L183: could you clarify why high concentrations lead to fewer monomers. Is that because endo-enzymes are busy breaking down large polymers and need time to make their way to monomers?

Yes, this is exactly the reason. We added a clarification to the manuscript, the line 240 now reads:

“ This delay stems from the fact that endo-enzymes cleave any bond within a polymer with equal probability. Consequently, there are numerous pathways to break down large molecules, most of which do not result in monomer production. In other words, the probability of generating monomers from long chains is extremely low when using endo-enzymes. However, as polymers gradually decrease in size as a result of degradation, the probability of generating monomers gradually rises. In essence, the reaction requires time to break down the polymers into progressively smaller chains, ultimately leading to the production of monomers, see lower panel of Fig. 4B.”

Supplementary information:
- Enzyme dynamics are described by a difference equation, while polymer mass balances are described by ordinary differential equations. I would homogenize the presentation of the equations, and explain how they are solved numerically (if using a finite difference scheme, were results numerically stable?).

We homogenize the presentation of the equations in the supplementary material. We also added a short paragraph about our numerical implementation, and the choices of time to guaranty the stability of the solution. We added a second paragraph to the Sec. C of the Supplementary Material, which reads:

“The temporal dynamics is advanced by a finite difference method. Finite differences are also used to implement spatial dynamics to model the diffusion process. The step size was adapted to guaranty the stability of both the diffusion and fragmentation processes, since numerical instabilities can lead to violation of conservation of mass in the system. We have chosen the values for dt from 0.05 to 0.005 depending on the set-up. The smallest dt values were used to simulation the dynamics with high diffusion coefficients.”

- I would suggest adding a table with all symbols defined and reporting their units. It would help understand equations that seem to have inconsistent units, like Eq. 9 where units should be mol/h per cell but the units on the right-hand side seem to be (interpreting the explanations in the text…) mol per channel per hour times channel area per cell. Channel number and channel area don’t have same units, so I could not recover the correct units of mol/h per cell.

We thank the referee for the suggestion and for noticing the inconsistency. We have added such a table to the supplementary material. We have also slightly changed the paragraphs around Eq.(9), to make all our derivations clearer, and correct for the inconsistencies.

Reviewer #2:

The main claim of the manuscript is that a trade-off emerges from biopolymer decomposition due to the differential dynamics of depolymerizing enzymes, or in other words, different depolymerization mechanisms have different ecological consequences. The originality of this finding, as far as I know, is quite high, and it has significant consequences to understand microbial ecological dynamics. Among others, I highlight the theoretical possibility of formation of consortiums under one single complex substrate, which expands the traditional view in microbial ecological dynamics that is usually examined in the light of Gause's (competitive exclusion) principle. This is just one example, and therefore, this manuscript has potentially general relevance in the field of microbial ecology.

However, I have some main comments and a few minor comments about your manuscript, which you can found below.

Introduction.
The detour that you take on the introduction from l. 34 and so on… well, I think you can easily lose readers there. The three paragraphs starting in line 34 have parts of introduction, methods, and even results. In my opinion, the different bits of information have to go to the relevant sections of the manuscript. I advise to state clearly the questions that you try to answer with the two surveys and add the background information in the methods (except the relevant information for the rest of computational approaches you followed, or the general discussion). I believe that you selected chitinases to inspire your computational research, so probably it would be better to introduce these three paragraphs after the mention to the gap of inclusion into models of the depolymerization process.

We sincerely appreciate the reviewer's feedback. In response, we have restructured the introduction to streamline the flow of the narrative. Specifically, we have eliminated redundancies with the Material and Methods section. However, we have chosen to retain the survey results in the introduction. We believe that providing an overview of the prevalence of endo- and exo-enzymes in microbial degraders is crucial for contextualizing our research question and the significance of our study. While we acknowledge that this structure may deviate from the conventional approach, it reflects a deliberate decision, and it's effectiveness was also positively received by the first reviewer.

Model.
I think that more detail should be included about the model. Without looking at references or the supplementary information, I can not decide if your model is deterministic/stochastic or in continuous/discrete time, neither I can see a clear description of the state variables. In my opinion, this makes less likely the reproducibility of this work. Please include more details in the main text.

In response to the referee's request, we have incorporated additional information about the model description into the main text. Specifically, we have introduced a new subsection titled "Model Overview" within the methods section, where we outline the main components and key processes considered in our model. However, due to the complexity of the model, certain details still remain in the Supplementary Material.

Additionally, I find that using reaction-based schemes to describe models, they are easier to understand, so I'd include it in the main text for all mechanisms in the model. For example, it is unclear to me whether your endo-enzymes are able to produce monomers. In fact, there's a mismatch between Fig. 1A and Fig. 2A, as they don't represent the same set of reactions. If endo-chitinases only produce dimers, and the chitobiase is needed for the absorption of monomers, then chitobiase should be modelled explicitly. The same can be said for exo-chitinases. An additional step in the metabolism may well change the dynamics of microbial growth.

Our manuscript aimed to highlight the impact of various depolymerization dynamics on microbial growth rather than specifically model the chitin degradation with all enzymes involved. We acknowledge that the mismatch between the two figures may cause confusion, therefore to ensure clarity we moved the Fig. 2A to the Supplementary Material and added an observation to its caption. To maintain a broader scope, we intentionally refrained from incorporating chitobiase, in the model. Moreover, as outlined in the introduction, chitobiase functions in the periplasm after cell uptake, and in this case cells have the capacity to uptake dimers as well. Introducing this additional step would only introduce unnecessary complexity to the model without altering the main claims of our manuscript. We also hope that the additional subsection with model overview will add clarity about processes included in the model.

Moreover, when I arrived to line 220 or so, I realize that in the methods there is no specifications on what are you going to look in with your, and you just introduce it in the results. I encourage you to make all your analyses explicit in the methods.

We thank the referee for the suggestion. In response, we have added a new subsection titled "Model Scenarios" at the end of the Methods section. This subsection provides a concise overview of the various scenarios analyzed in the manuscript. We opted to keep this section succinct to avoid unnecessary repetition and avoid overlapping with the results section.

Finally, some minor things. I think there is a need to justify why do you allow only a single type in your grid cells, and why do you divide biomass by half. Does your results change in you keep change the proportion of biomass in the new cell vs the original one?

We assume that within a grid cell the system is well mixed (as explained in the methods) since all microorganisms are very close to each other and exchange of products is very fast. Introducing the coexistence of two populations within a grid cell would negate the spatial effects we aim to capture. In such a scenario, the two species would have access to both enzyme types and behave in principle as members of a single generalist species. To clarify the issue we added a sentence at the line 173:

“ *Enforcing the condition of only one type of microorganism per microsite allows us to model consortia in which each microorganism type has preferential access to the degradation products of the enzyme it produces.”*

The division of biomass in half was chosen to avoid introducing undue complexity or assumptions to our model about the population spreading. The dynamics of spreading of the population may slightly change in case of a different choice for the division of biomass. The “daughter” cells would contain less biomass and take longer to divide, while the “original” site will reach the division threshold sooner. Based on our results a small population could be a disadvantage for endo producers, that need to wait on enzyme degradation products, while their small population may rapidly collapse. Although these aspects are interesting extensions of our results, we do not want to extend further our analysis, and focus only on what we think are the core aspects of the system.
We also clarify this issue in line 178 of the text:

“When microbial biomass within a microsite exceeds a certain limit, the biomass is divided and part of it is transferred to the neighboring site. Although for simplicity we assume that biomass is divided in half, it is important to recognize that altering the division method may lead to slight changes in the dynamics of population spreading.”

Use of the term trade-off.
I am not so sure that microbes are facing a trade-off here, as generalist are predominant. What is the trade-off here? Ecology has compelling examples, like competition versus colonization, or longevity vs fecundity. But in your case, I struggle to find the competing processes. In the end, this is just a terminology problem but it may lead to some ecologists to confusion. It would be great if you could clarify the "trading-off" elements, but I would prefer too call it alternative strategies. In any case, I really like Table 1 (except for the mention to trade-off, as you could understand).

We disagree with the referee on this point. Given that a bacteria has a finite amount of resources it experiences a trade-off to invest in one or the other enzyme. In other words an increase in the production of one enzyme means a decrease in the production of the other, since the total amount of carbon allocated is finite. What our model analysis shows is that, driven by the inherent physical interaction of the two enzyme types with their substrate, enzyme-producing bacteria can either be superior at low substrate concentrations (when they produce endo-enzymes) or at high substrate concentrations (when they produce exo-enzymes). We do not think that trade-off implies that the two competing process are exclusive and cannot overlap, it just implies that increase of one process implies a decrease in the other. We also do not think that the trade-off negates that could be an optimum ratio of the two enzymes produced, which is what we actually find.

The term trade-off is broadly used to characterize general bacterial traits, here are examples:

1. Ramin & Allison (2019), *Front. Microbiol*. 10:2956. doi: 10.3389/fmicb.2019.02956 : analyse the tradeoff between growth and enzyme production. These two processes are obviously not exclusive, but an increase in one means a decrease in the other.

2. Malik et al *ISME J* **14**, 1–9 (2020). https://doi.org/10.1038/s41396-019-0510-0: also reviews the tradeoff between microbial investment into growth, enzymes or defense strategies

We agree with the referee that the Table 1 does not exactly represent the trade-offs, therefore we used reviewers suggestion and it now reads:

"Advantages and downsides of alternative strategies of microorganisms in degradation of complex substrates."

For clarity we added a summary to last paragraph of the discussion, where we can fully discuss the trade-off we uncover line 447, now reads:

"In summary, considering that bacteria have a finite amount of resources, investing in one enzyme necessarily reduces the investment available for the other. In this context our model analysis demonstrates an intrinsic trade-off: that enzyme-producing bacteria can excel either at low substrate concentrations (by producing endo-enzymes) or at high substrate concentrations (by producing exo-enzymes), driven by the inherent depolymerization dynamics of the two types of enzymes. As a consequence we show that bacteria would have to optimize the two ratios to survive in both conditions."

Copiotrophs and oligotrophs.
The paragraph going from line 307 to 323 adds confusion to the manuscript. I do not recall mentions of copiotrophs and oligotrophs until this moment in the manuscript, and as you say there is no exact agreement with the traditional view of these terms. So, I think you can skip this entire paragraph, for the sake of clarity and a simpler train-of-thought.

We agree with the referee, we have completely rewritten this paragraph to clarify what we mean, and taking away this particular classification. This section of text now reads, see line 345:

"In summary, our results show that different types of environments would favor the producers of one or the other enzyme: oligotrophic environments favoring endo-producers and copitrophic ones favoring exo-producers. They also suggest that in a succession dynamics, the exo-producers could establish the initial population in an environment rich with complex substrate, followed by endo-producers when the substrate becomes depleted."

Minor comments.
l. 11 Bacteria can not assess the expected return of an investment. Please delete the phrase and rephrase l. 215 and l. 397 too.

What we meant by our statement is that the bacteria spend energy in enzyme production with the expectation of later increasing their uptake rates, which would constitute a somehow an indirect "assessment" of such investment. In this view if there is no increase in the uptake, the resources used in enzyme production were wasted. In other words the decision to allocate energy towards enzyme production is indeed an investment for bacteria, with growth outcomes depending on its effectiveness.

However we agree with the reviewer that it is unclear how a direct "assessment" of this investment could be made by bacteria. Therefore to avoid misunderstanding, we completely avoid this formulation. We rephrase the old l.11 (new line 9), which now reads:

"On the other hand, the production of each additional enzyme adds to the metabolic costs of microorganisms."

We also slightly modified the old l.215 (new line 249) to read:

"In other words there is an intrinsic time period during which the microbes are able to wait for the appearance of monomers."

and also the old l.397 (new 437):

"We can only speculate that these microorganisms inhabit environments that offer alternative nutrient sources that enable endo-producers to endure while awaiting an for increase in their uptake rates."

l. 21-25 This sentence is too long and not so clear. Please reformulate, maybe cutting it in half.

We thank the referee for pointing this out. The exert now reads:

"Hehemann et al showed evidence that through adaptive radiation a population of closely related bacteria has subdivided into specialized ecophysiological types adapted to the given environmental opportunities. These results showed that there is a great flexibility and variability of the depolymerization pathways and hint to the existence of trade-offs for the production of the enzymes engaged in depolymerization."

l. 28 It is the first time that as a community ecologist I encounter the EC 3.2.1.* nomenclature. The reader might need to see Enzyme Class (EC) before adding directly the abbreviature. Moreover, the example provided is odd, you mention enzymes and give an example of substrates such as cellulose, which is no an enzyme. In this case, I'd be more verbose.

The sentence now reads:

"… glycoside hydrolases (Enzyme Class (EC) 3.2.1., e.g. for substrates such as cellulose and chitin) or other lyases (e.g. for substrates such as alginate)."*

l. 334 This is your third hand. Please rephrase.

The paragraph was updated.

l. 324-353 Please discuss whether your diffusion mechanism is similar or not to the actual situation in soils. I would say that in soils there are pulses of diffusion (caused by rain) rather than a constant one.

Please notice that we model a process at a small scale and that spans a relatively short time period. In the case of a rain event, we anticipate advective loss of small substrates and even passive advection of microorganisms, processes which we do not consider in our model. Instead we focus on the time between these events where there are no changes in soil water content. To enhance clarity, we have provided the following explanation to line 385:

"It is important to note that our model considers the dynamics at very small spatial (the grid cell is 10 μm^3) and relatively short time scales. The diffusive exchange we model, both between and within

micro-habitats, is tied to connectivity of the soil's water film. The number and size of aqueous bacterial habitats would change with the soil water content, which would follow the drought or rainy periods.”

l. 372-398 I find this paragraph too speculative. Please anchor it better in the literature or just delete it.

We have shorten and rephrased the paragraph, we also added an explicit statement about the speculative nature of some of the statements within it.

l. 399-407 I am sure that you can do a much more convincing conclusion than this one. Why is your manuscript relevant? What are your biological insights? Fly high.

We thank the referee for the incentive, we extended the final paragraph the new part starts on line 454 and reads:

“Finally, our theoretical analysis illustrated that microbial consortia can form in the degradation of a single complex substrate, by splitting the production of apparently similar but complementary enzymes. We also showed how interactions within such consortia can change from positive to negative depending on substrate availability. Such context dependent interactions broaden our understanding of the dynamic nature of microbial communities and can be used to expand our microbial population models (Lotka-Voltera or Resource-Consumer models) to account for such effects.”

- There are typos in Figure 5 A and C.

We thank the referee for noticing, we have corrected the typos.

- Figures are in general too small and with many panels. Maybe you could consider to send some to the supplementary and make the rest a bit bigger.

We thank the reviewer for the suggestion. We modified Fig. 2 and 5 to improve visualization. In particular we moved the panel A from Fig.2 to the supplementary material as mentioned previously.

- The github code for the first part needs more explanation in the read.me. It should be clear how to run the code in both repositories. Not all ecologists know Python (R is much more common for ecologists).

We updated the Readme file with some instructions about python installation and additional code description.

- Maybe you could discuss how including explicit mechanisms of polymer degradation or consumption of resources changes ecological insight into the dynamics of microbial communities. E.g. https://doi.org/10.1007/s12080-020-00466-7

We have expanded the last paragraph of the discussion section of the paper to include the possible implications of our results for microbial community dynamics and included the citation.
