# Supplementary material for: Bacteria face trade-offs in the decomposition of complex biopolymers
Source: PLoS Comput Biol. 2024 Aug 8;20(8):e1012320. doi: 10.1371/journal.pcbi.1012320 (PMC11364420; doi:10.1371/journal.pcbi.1012320)
Supplement: Attachment — Submitted filename: response_letter_2.pdf [file pcbi.1012320.s004.pdf]

Dear Tobias Bollenbach,

Thank you for the referee reports, which we have carefully taken into account. In particular we have restructured the introduction according to suggestion of Reviewer 2. We thank both referees for their very positive and constructive comments. Our detailed reply to each reviewer's comments (quoted from the report and stated in bold) including a description of main changes we made to the manuscript and supplementary material are given below.

On behalf of the authors,

Ksenia Guseva

##

Reviewer #1

We thank the reviewer for carefully checking the manuscript and the supplementary material again, and for noticing minor inconsistencies. We hope that in this version we managed to address all of them.

I have a few remaining minor comments and one technical question. Starting from the latter: could you provide some more explanations regarding Eq. (6) in the Supplementary materials? As written, it does not depend on i, and for any j>1 we get g<1, which is strange since g is defined as the number of molecules of size i (not a fraction).

We thank the reviewer for noticing this inconsistency, g actually is defined as the probability (not "the number") of a molecule of size i to be produced by hydrolysis of a molecule of size j. We have corrected this mistake in the new version.

Moreover, j-1 simplifies away when plugging Eq. (6) into Eq. (7).

Exactly, this term works to simplify with the (j-1) of the f' function (Eq. 5) in the second left-side-term (production of i-mer from all other fragmentation events). However notice that the (j-1) term is still present in the f' as it enters the first left term of the equation (the negative term), because there we need all the ways a molecule of size i can be broken.

It is easier to understand with an example. For simplicity please consider the case without Michaelis-Menten kinetics. Lets take a 3-mer (i = 3). It can be broken in i-1 = 2 ways: 2+1, 1+2 (second term on the left side), so this should be accounted in the negative term. In addition it can be produced by breaking all other larger polymers. The Eq. (5) for i=3 reads:

$N_3 = N_3 - (2)*N_3 + (A*N_4 + B*N_5 + C*N_6 + D*N_{7+...})$.

Now we need to determine the coefficients A, B, C, D and so on. For that we need to take into account how many 3-mers are produced from the fragmentation of each j-mer (j >i):

4-mer: 1+3, 2+2, 3+1 → A = 2 ways to get 3-mers

5-mer: 1+4, 2+3, 3+2, 4+1 →B = 2 ways to get 3-mers

6-mer: 1+5, 2+4, 3+3, 4+2, 5+1 → C = 2, 1 way to get two 3-mers

7-mer: 1+6, 2+5, 3+4, 4+3, 5+2, 6+1 → D = 2 ways to get 3-mers

Notice that we do not need to know how each one of these polymers may be broken, since we only need 3-mers. Therefore we do not need the j-1 in the positive term. In the second term the function g helps to control for that. In summary the interplay of g and f' functions guaranty that there is no loss of mass (monomers) in the system. Please note that this conservation was also checked when our simulations were tested.

Main text
L150: not clear what 'label substrate' means

There was a typo, we meant "labile", but for clarity we changed the text to "monomers".

L163: N eliminated from the cell would accumulate as mineral N?

The eliminated N re-enters the DIN pool, we added a clarifications into the text, now the lines 139-140 read:

"The eliminated nitrogen re-enters the DIN pool."

L164: a lower limit for cell biomass?

We have added to the text that the minimum biomass per microsite can reach is 0.08 mol C. Now the new line 142 reads:

" We set up a minimum biomass per microsite (0.08 mol C), after which the microorganism is considered dead."

L357-359: the reason why oligomers are lost and not used on site is explained around L405, but it would be useful to explain it here already

We added the sentence to clarify the issue, now the new lines 380-383 reads:

"This is because depolymerization dynamics of endo-enzymes produces a high number of intermediate products, these oligomers are also subjected to diffusion but cannot yet be taken up by the cell. "

Supplementary materials

We thank the referee for all the minor corrections, we included them all in the text. Here we add our response to the questions posed by the referee.

Several symbols are not included in Table S4; e.g., U_{cell}, n_{col}, C_b, dt, k_1

We added the missing symbols to the table.

Eq. (11): what is the difference between U_{cell} and U_{pot}?

We apologize for the confusion both represent the same variable. We forgot to fully change the notation from U_{pot} to U_{cell} everywhere in the text. We corrected the mistake in the current version.

Eight line in Subsection (vi): I am not sure I understand—when there are three cells, the colony is split in half (1.5 cells?) and one half is moved to nearby grid cells? Or when reaching a number of four cells, so the colony is split in two small colonies of two cells each?

For a division to occur the biomass in a microsite has to exceed that of 3 cells of average size. In other words, when algorithm identifies 4 or more cells in a microsite, it tries to divides them into two colonies (if there is a free space). The biomass is then indeed divided into two identical parts (even if the colony has an estimated uneven number of members). Note that in our approach we work with the biomass and the cell number within a microsite is only an estimation. In other words the model does not track how exactly the biomass is spread among the cells within a microsite when the division occurs. We consider this to be a reasonable assumption, since the cells must not have identical sizes within colonies.

We reformulated this part and give some additional information, and the text now reads:

" We assume that a microsite-colony can contain at most the biomass of 3 average-size cells (Bmax = 3) without attempting to spread in space. Once the biomass of a microsite exceeds 12 mol C/MS (indicating the presence of 4 cells or more), the colony would divide. Note that the algorithm does not track how the biomass is exactly divided within the members of a single micro-site colony, and the number of members within this colony is only an estimation based on the average-cell size provided."

Also, this threshold value for colonization of nearby cells seems much smaller than the theoretical maximum of 1000 cells reported above

This value is indeed much smaller than the 1000 cells that would fit if tightly packed into the microsite. However, as explained in the text, we assume that there are also other components in the system filling the microsite. It is important to note that the microsite-colony can grow beyond the given limit if there is no place to spread (all neighboring sites are occupied).

We have observed that the growth of the population, especially of endo-producers, is affected by the minimum size necessary for them to spread. Moreover, we have observed an interplay between this parameter and the diffusion rate of oligomers, which has a non-trivial effect on the survival of populations, suggesting trade-offs between staying or spreading in space. However, we find that this exploration goes beyond the scope of the current study and also does not affect the nature of our current findings.

Fig. S3: how is the presence of soil matrix accounted for in the definition of D_n? generally diffusivities in pore water are scaled by soil saturation level (or porosity if the soil is saturated)

Unfortunately we did not take into account the porosity of the soil matrix for D_n calculation, only how the diffusion coefficient would scale down with the molecular weight. This indeed may further slow down the diffusion of oligomers. We added this observation to the caption of Fig.S3, which now reads:

" Note that the scaling down of D_n accounts only for how the coefficient changes with molecular weight of the polymers, however the porosity of the soil matrix may further slow down the diffusion rates."

###

Reviewer #2:

We thank the reviewer for his positive and very constructive comments. We made additional effort to improve the structure of the introduction in agreement to his last detailed suggestions and hope that this makes our work more easily accessible and it attracts a wider audience.

Minor details

By the way, you talk about Figure 2D, that is not actually present in the text. I believe that your present references to Figures 2B, C and D should be actually 2A, B and C, because you moved old figure 2A to the supplementary.

Thank you for noticing. Corrected.

You have Enzyme Classes and Enzyme Commission number for the same abbreviation (EC). I believe that the first appearance should be Enzyme Commission class. I got a bit confused when I saw both having the same abbreviation.

Corrected.

Finally, the term copiotrophic applied to environments is unusual, because it implies a specific strategy of the organisms. I think the readers would be more fond of the term carbon-rich environment instead.

Corrected.
